# Progression of carotid plaque burden in patients with polycythemia vera and essential thrombocythemia

**DOI:** 10.1007/s44313-025-00098-y

**Published:** 2025-09-01

**Authors:** Seong Soon Kwon, Sun Young Jeong, Min-Young Lee, Kyoung Ha Kim, Namsu Lee, Jong-Ho Won, Seug Yun Yoon

**Affiliations:** 1 Division of Cardiology, Department of Internal Medicine, Soonchunhyang University Seoul Hospital, Seoul, Korea; 2 Division of Hematology & Medical Oncology, Department of Internal Medicine, Soonchunhyang University Seoul Hospital, 59 Daesagwan-Ro, Yongsan-Gu, Seoul, 04401 Republic of Korea

**Keywords:** Essential thrombocythemia, Polycythemia vera, Carotid plaque burden

## Abstract

**Purpose:**

Prevention of vascular events is the main objective in patients with polycythemia vera (PV) or essential thrombocythemia (ET). Carotid ultrasonography (USG) is a safe and noninvasive diagnostic tool that can be used to stratify cardiovascular and stroke risks. In our previous study, carotid plaque burden was significantly higher in patients with PV/ET than in the general population. This study aimed to determine changes in carotid plaques in patients with PV/ET.

**Methods:**

We retrospectively evaluated the medical records of patients with ET/PV who had undergone carotid USG at least twice.

**Results:**

Of the 56 patients, 30 had PV and 26 had ET. The carotid plaque score was increased in the follow-up carotid USG compared with that in the initial carotid USG (3.38 ± 1.47 vs. 3.73 ± 1.46, *p* = 0.0139). The carotid plaque burden at the time of follow-up carotid USG showed no significant differences in patients with a complete hematologic response (CHR); however, it significantly worsened in patients who failed to achieve CHR.

**Conclusion:**

We confirmed that the carotid plaque burden persisted during follow-up in patients with PV/ET. A CHR may prevent an increase in carotid plaque burden.

**Supplementary Information:**

The online version contains supplementary material available at 10.1007/s44313-025-00098-y.

## Introduction

Myeloproliferative neoplasms (MPNs) are chronic clonal disorders associated with persistent inflammation driven by malignant hematopoietic clones. In essential thrombocythemia (ET) and polycythemia vera (PV), this inflammatory milieu may contribute to the progression to myelofibrosis or acute leukemia. Although these advanced stages require intensive therapy, the primary goal of PV/ET management is to prevent thrombotic events, including myocardial infarction and stroke. Risk-adapted use of low-dose aspirin and cytoreductive agents is commonly employed for this purpose.

Carotid ultrasonography (USG) is a noninvasive modality that enables early detection of subclinical atherosclerosis and stratification of cardiovascular risk. In our previous study, we reported significantly higher carotid plaque scores in patients with PV/ET than in propensity score-matched controls [1]. In this study, we sought to evaluate the progression of carotid plaque burden over time in patients with PV/ET.

## Methods

### Study population

We retrospectively reviewed the medical records of patients diagnosed with PV or ET based on the World Health Organization 2016 criteria between January 2008 and February 2023 at Soonchunhyang University Seoul Hospital. Patients who underwent carotid USG at least twice, with an interval of ≥ 6 months, were included. Complete hematologic response (CHR) was defined as hematocrit < 45% without phlebotomies, platelet count < 400 × 10^9^/L, and leukocyte count < 10 × 10^9^/L [2]. Patients were categorized into CHR and non-CHR groups based on follow-up laboratory values at the time of the second carotid USG, and changes in carotid plaque scores were compared within and between the groups. This study was approved by the Institutional Review Board of Soonchunhyang University Hospital (IRB No. 2024–08-003). The requirement for informed consent was waived because of the retrospective nature of the study.

### Carotid USG examination

Carotid USG was performed by a single experienced sonographer, following the protocol used in our previous study [1].

### Statistical analysis

Categorical variables are presented as numbers (percentages) and compared using the chi-square or Fisher’s exact test. Continuous variables are expressed as mean ± standard deviation and compared using Student’s t-test. Pearson correlation was used to assess the relationship between changes in carotid plaque scores. Statistical significance was set at *p* < 0.05. All analyses were performed using R version 3.6.1 (R Foundation for Statistical Computing, Vienna, Austria) and Rex version 3.0.3 (RexSoft Inc., Seoul, Korea).

## Results

### Study population

A total of 56 patients were included: 30 with PV and 26 with ET. The median interval between carotid USG examinations was 18 months (range, 5–24 months). Eighteen patients were newly diagnosed during the observation period. The baseline clinical and demographic characteristics are summarized in Table 1. Among all the patients, 17 (30.4%) had diabetes, 37 (66.1%) had hypertension, and 31 (55.4%) had dyslipidemia. Lipid-lowering agents were used in 32 patients (57.1%), and the mean low-density lipoprotein (LDL) cholesterol level was 81.6 ± 27.3 mg/dL.

**Table 1:** Baseline characteristics of study population

	Total (*n* = 56)	PV (*n* = 30)	ET (*n* = 26)	*p* value
Age (years)	67.09 ± 12.27	66.1 ± 12.06	68.23 ± 12.64	0.5232
Male	31 (55.36%)	20 (66.67%)	11 (42.31%)	0.1189
Current smoker	6 (10.71%)	2 (6.67%)	4 (15.38%)	0.5738
BMI	25.42 ± 4.03	25.57 ± 3.49	25.25 ± 4.67	0.777
JAK2 mutation	47 (83.93%)	27 (90%)	20 (76.92%)	0.335
Comorbidities				
Diabetes	17 (30.36%)	10 (33.33%)	7 (26.92%)	0.8189
Hypertension	37 (66.07%)	22 (73.33%)	15 (57.69%)	0.3421
Dyslipidemia	31 (55.36%)	15 (50%)	16 (61.54%)	0.5507
Lipid-lowering agent	32 (57.14%)	15 (50%)	17 (65.38%)	0.3737
Laboratory data				
WBC (10^3^/µL)	10.49 ± 6.44	12.19 ± 8.13	8.53 ± 2.66	0.0259
Hb (g/dL)	14.33 ± 2.18	15.25 ± 2.3	13.27 ± 1.44	< 0.001
Platelet (10^3^/µL)	491.27 ± 260.49	401.40 ± 281.81	594.96 ± 190.48	0.0037
Glucose (mg/dL)	118.57 ± 32.17	116.6 ± 28.4	120.85 ± 36.5	0.6331
Total cholesterol (mg/dL)	151.48 ± 36.07	157.3 ± 36.16	144.77 ± 35.47	0.1969
LDL cholesterol (mg/dL)	81.58 ± 27.3	85.73 ± 28.75	76.6 ± 25.1	0.2142
CRP, mg/L	0.12 ± 0.12	0.15 ± 0.13	0.09 ± 0.08	0.0386
N/L ratio	4.37 ± 5.27	5.87 ± 6.81	2.64 ± 1.26	0.0161
Carotid USG				
Mean IMT (mm)	0.65 ± 0.11	0.67 ± 0.11	0.63 ± 0.1	0.2252
Plaque score	3.38 ± 1.47	3.37 ± 1.56	3.38 ± 1.39	0.9639

*BMI* body mass index, *ET* essential thrombocytopenia, *Hb* hemoglobin, *IMT* intima-media thickness, *JAK2* Janus Tyrosine Kinase 2, *N/L* neutrophil–lymphocyte, *LDL* low-density lipoprotein, *PV* polycythemia vera, *CRP* C-reactive protein, *WBC* white blood cell

### Carotid plaque progression during follow-up

**Fig. 1:**
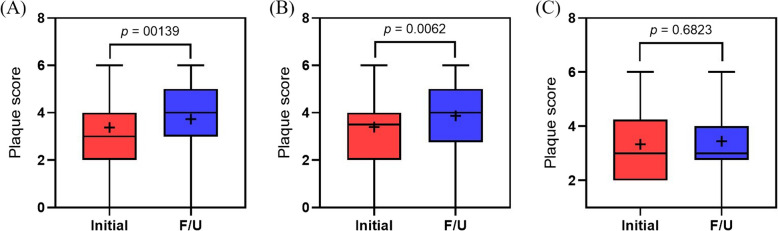
Comparison of baseline and follow-up carotid plaque score. **A** Total population, **B** non-CHR group, **C** CHR group. CHR, complete hematologic response

The carotid plaque score increased significantly at follow-up compared with that at baseline (3.38 ± 1.47 vs. 3.73 ± 1.46, *p* = 0.0139). In patients who achieved CHR (*n* = 18), no significant change in plaque burden was observed (3.33 ± 1.41 vs. 3.44 ± 1.34, *p* = 0.6823). Conversely, in patients who failed to achieve CHR (*n* = 38), plaque scores worsened significantly over time (3.39 ± 1.52 vs. 3.87 ± 1.51, *p* = 0.0062) (Fig. 1).

To assess whether CHR was independently associated with plaque progression, we performed a multivariate logistic regression analysis after adjusting for age, sex, diabetes, hypertension, and baseline intima-media thickness. CHR achievement remained a significant independent predictor of reduced risk of plaque progression (odds ratio = 0.099, 95% confidence interval: 0.012–0.808, *p* = 0.0399) (Supplementary Table S1).

## Discussion

To the best of our knowledge, this is the first longitudinal study to assess carotid plaque progression in patients with PV and ET. We observed a significant increase in carotid plaque burden during follow-up, suggesting that MPN-associated inflammation contributes to ongoing atherosclerosis despite treatment.

While previous studies have suggested that statins reduce thrombotic risk in patients with PV/ET [3], more than half of our patients were receiving lipid-lowering agents; however, the plaque burden still progressed, even with controlled LDL levels. Plaque progression was not observed in patients who achieved CHR, whereas those who failed to achieve CHR showed significant worsening, highlighting the potential role of hematologic control in mitigating vascular risk. In PV, maintaining a hematocrit of < 45% is known to reduce cardiovascular events [4]. Maintaining appropriate levels of white blood cells and platelets is also important because elevations in hematocrit, platelet count, and leukocyte count are associated with an increased risk of thrombosis [5]. Among these parameters, leukocytosis contributes to thrombotic events and disease progression [6, 7]. In patients with ET, careful control of leukocyte and hematocrit levels, often through modulation of platelet count, remains a key therapeutic consideration [7].

Although no thrombotic or cardiovascular events occurred during the follow-up period, the observed increase in carotid plaque burden may reflect subclinical vascular progression. Carotid plaque burden is a validated surrogate marker for atherosclerotic cardiovascular disease risk, and its worsening over time warrants clinical attention. Nevertheless, the absence of clinical event data was a key limitation of this study.

This study had several limitations. The retrospective design, small sample size, and variability in follow-up intervals between carotid USG assessments may have influenced the detection and degree of plaque progression. In addition, the relatively short follow-up duration may have been insufficient to capture overt vascular events. These factors limit the generalizability of our findings and highlight the need for larger prospective studies with standardized and extended follow-up periods.

### Conclusions

Carotid plaque burden increased over time in patients with PV and ET, despite statin use and controlled LDL levels. Notably, plaque progression was not observed in patients who achieved CHR, suggesting that effective hematologic control helps prevent atherosclerotic progression in MPNs.

## Supplementary Information

Supplementary Material 1.

## Data Availability

No datasets were generated or analysed during the current study.
