# Supplementary material for: Progression of carotid plaque burden in patients with polycythemia vera and essential thrombocythemia
Source: Blood Res. 2025 Sep 1;60(1):48. doi: 10.1007/s44313-025-00098-y (PMC12401794; doi:10.1007/s44313-025-00098-y)
Supplement: Supplementary file 1 — Supplementary Material 1. [file 44313_2025_98_MOESM1_ESM.docx]

## Supplementary Table 1. Multivariate logistic regression analysis for predictors of carotid plaque progression

| Variable | Estimate | Odds Ratio | 95% CI for Odds Ratio | SE | Z-value | p-value |
| --- | --- | --- | --- | --- | --- | --- |
| CHR achievement | -2.317 | 0.099 | 0.012 – 0.808 | 1.074 | -2.158 | 0.0399 |
| Hypertension | 2.433 | 11.391 | 1.703 – 76.007 | 0.964 | 2.512 | 0.0120 |
| Diabetes mellitus | -2.654 | 0.070 | 0.004 – 0.695 | 0.998 | -2.660 | 0.0099 |
| Sex (male vs. female) | -1.429 | 0.239 | 0.051 – 1.117 | 0.786 | -1.818 | 0.0690 |
| Age (per 1 year) | -0.047 | 0.954 | 0.896 – 1.016 | 0.032 | -1.460 | 0.1441 |

Abbreviations: CHR, complete hematologic response; CI, confidence interval; SE, standard error; IMT, intima-media thickness.
Note: IMT was included in the model as a covariate but not shown due to collinearity concerns or lack of statistical contribution in the final model.
